# Midterm Results of Cementless and Cemented Unicondylar Knee Arthroplasty with Mobile Meniscal Bearing: A Prospective Cohort Study

**DOI:** 10.2174/1874325001711011173

**Published:** 2017-10-31

**Authors:** Radosław Stempin, Wiesław Kaczmarek, Kacper Stempin, Julian Dutka

**Affiliations:** 1 Department of Orthopedic Surgery Promienista Clinic, Poznan, Poland; 2 Department of Orthopedic & Trauma Surgery Westallgäu Clinic, Wangen, Germany; 3 Department of Orthopedic& Trauma Surgery S. Zeromski Memorial Hospital Cracow, Poland

**Keywords:** Osteoarthritis, knee, Unicompartmental knee arthroplasty, Mobile meniscal bearing, Cohort study, Implant fixation, Clinical outcome, Bone hardness test

## Abstract

**Background:**

Cemented unicompartmental knee arthroplasty (UKA) yields good clinical outcome but common revision reasons are loosening and pain. Cementless UKA may reduce the revision rate.

**Objective:**

The current study was designed to assess clinical and radiographic outcome of cemented and cementless UKA, using bone quality as determined by the Bone Hardness Test (BHT) as selection criterion for cementless implantation.

**Methods:**

In this prospective comparative cohort study we analyzed 50 cementless and 29 Oxford consecutive UKA cases. Patients with sufficient bone quality were eligible for cementless UKA. Bone quality was assessed with the BHT, which consisted of exercising pressure with the thumb on the bone surface created after resection of the tibia.

**Results:**

The average surgical times were 62.5 ± 12.6 and 78 ± 16 minutes in the cementless and the cemented group, respectively (p < 0.01). The average thickness of the polyethylene insert was 4.3 ± 1.2 (range, 3 – 9) and 3.7 ± 0.8 (range, 3 – 6) mm, respectively (p = 0.02). Both types of implants yielded excellent clinical and functional results. At an average follow-up time of seven years, we found non-significant differences between clinical results of cementless versus cemented implants.

**Conclusion:**

Shorter surgical time makes cementless implantation more attractive to surgeons when considering UKA options for their patients. The average thickness of the polyethylene insert in cementless group was 0.6 mm thinner than in the cemented group. The BHT is a simple and useful test to assess whether patients are eligible for cementless UKA.

## 1 INTRODUCTION

The Oxford unicompartmental knee arthroplasty (UKA; Zimmer-Biomet, Bridgend, UK) is a frequently used treatment of medial compartment knee osteoarthritis, and is used less commonly in lateral compartment affection. UKA is a unique implant containing a mobile meniscal bearing fully adapted to both spherical femoral and flat tibial components. John Goodfellow first used UKA in 1976 (Phase 1) [1]. The insert reduces polyethylene wear and eliminates tangential forces acting on the tibial surface, thus improving implant survival [2-6]. The first clinical use of the Oxford UKA occurred in 1982 [1]. Surgeons first used a spherical reamer in 1987 (Phase 2) [7], and since 1998, five sizes of femoral components and left and right sided tibial components have been available for clinical application (Phase 3). Cementless implants (with femoral compartments using a twin peg design) arrived in 2003. UKA in properly selected patients is a less invasive procedure than total knee arthroplasty (TKA) and closer to natural knee kinematics because the cruciate ligaments are retained, thus improving the functional results of surgery [5, 6, 8, 9]. The advantages of UKA include less blood loss during surgery, a lower complication rate, and a lower cost of the implant [10]. Also, revision procedures are easier in UKA cases than in TKA cases. Designers of the Oxford UKA phase 3 reported excellent survival rates of the prosthesis, reaching 98% at the ten-years follow-up and 96% when considering all related revision cases in the same follow-up period [11]. Independent third-party studies reported slightly inferior results, with ten-year follow-up survival reaching only 83% to 90%. These differences may have resulted from a learning curve problem debated in the literature. Centers with higher caseloads and experience with UKA have better results than centers using UKA less frequently [12]. In 2005, the Swedish Knee Arthroplasty Register reported a longer survival for third generation implants compared with second generation implants [13]. In February 2013, a published study analyzed the results of six years’ follow-up of 1000 cementless Oxford UKA phase 3 implants and reported a 97.2% survival rate [14]. Porous coated titanium surface covered with hydroxyapatite provided excellent osseointegration. A prospective randomized comparative study reported a markedly reduced rate of radiolucent lines (RLLs) below the tibial component in cementless implants [11, 15].

The current study presents clinical and radiographic outcome of cemented and cementless UKA, using bone quality as determined by the Bone Hardness Test as selection criterion for cementless implantation.

## 2 MATERIAL AND METHODS

In this prospective cohort study, we compared the clinical results of 79 consecutive minimally invasive operations using unicondylar cemented and cementless Oxford phase 3 implants from 2009 to 2010 for patients with knee osteoarthritis. To be included in the study, patients had to have primary medial compartment osteoarthritis with a well-preserved lateral compartment with competent cruciate ligaments and correctible intraarticular varus deformity (*i.e.*, the same criteria for Oxford UKA implant use [11]). Additional criteria were fixed flexion deformity of < 15°, flexion up to 110° under anesthesia and pain on the medial joint line. The degree of degenerative changes anteriorly, body mass index, age, sex, and bone mineral density did not affect the surgical decision. Patients with fixed varus deformity > 15°, inflammatory arthritis, previous high tibial osteotomy or ACL reconstruction were excluded [11]. In addition, patients with severe arthritis of the lateral facet of the patella were not eligible for the procedure [16]. The baseline characteristics of the study cohorts are presented in (Table **1**).

Two patients with cemented implants and one patient with a cementless implant died due to unrelated causes during the course of the study; these patients were not included in the study.

Good stability and good bone quality were the essential criteria for implantation of the cementless UKA. Ligament stability was evaluated by verifying the tension in the ACL after it had been visualized intraoperatively. The decision whether to cement the implant was based on the results of an intraoperative visual inspection of bone quality, and on a Bone Hardness Test (BHT). Exercising pressure with the thumb (or the index finger in case of a small knee) on the surface created after resection of the tibia allowed the surgeon to assess the hardness of bone tissue. If the pressure exerted on the bone caused the deflection of resected surface (*i.e*., if the thumb delved into the bone tissue), the hardness of bone was not deemed to be sufficient to provide primary stability of the implant, and a cementless implant was not used. Based on the outcome of this test, 29 cases received the cemented version of the Oxford phase 3 implant, and 50 cases were deemed eligible for the cementless version. We based clinical results on initial preoperative clinical assessments and final postoperative results. The average follow-up period for the cemented implant group was 7.3 ± 0.4 (range, 6.8 to 7.5) years, and the average follow-up period for the cementless implant group was 6.5 ± 0.2 (range, 6.2 to 7.2) years.

The two senior authors (R.S., W.K.) performed all procedures. A clinical assessment and standing radiography provided the basis for the primary preoperative decision for surgery using an Oxford UKA implant. The final decision depended on an intraoperative assessment of ligamentous stability and tactile probing of bone quality. Most surgeries used epidural anesthesia and a few used general anesthesia. All surgeries used a minimally invasive anteromedial approach, minimized bone resection, and a tourniquet [17, 18]. Patients used routine anti-deep venous thrombosis prophylaxis for 14 days starting from the operation day, except for patients with an increased risk of thromboembolic complications (such as those with varicose veins of the lower limbs, or a history of deep-vein thrombosis or pulmonary embolism). In these cases, we extended anti-deep vein thrombosis prophylaxis for 30 days after surgery. Patients used prophylactic perioperative antibiotic therapy for three days. Early rehabilitation including knee exercises and protected weight bearing started within the first 24 hours after surgery.

We assessed clinical outcomes using the Knee Society Score (KSS) [19], the Western Ontario and McMaster Universities Osteoarthritis Index (WOMAC) [20], and the Oxford Knee Score (OKS) [21]. Due to the observational nature of the study, patients were not examined radiographically at final follow-up. However, a substantial number of patients (cemented, n = 23 knees; cementless, n = 38 knees) brought their own radiographs at final follow-up, which were taken by their general practitioner. All available radiographs were examined for the presence of RLLs, implant loosening, and migration.

Continuous data are presented as the mean ± standard deviation (SD) and range. Categorical variables are presented as frequencies and percentages. To test for between-group differences, the Fisher exact test was used for categorical variables and the Student’s t-test for continuous variables. MedCalc 17.6 (MedCalc, Ostend, Belgium) was used for statistical analysis.

## 3 RESULTS

We found a significantly shorter average surgical time in the cementless group (62.5 ± 12.6 (range, 35 - 95) minutes) compared with the cemented group (78 ± 16 (range, 50 - 115) minutes) (p < 0.01). The average thickness of the polyethylene insert was 3.7 ± 0.8 (range, 3 – 6) cm in the cementless group and 4.3 ± 1.2 (range, 3 – 9) cm in the cemented group (p = 0.02). In the follow-up observation, mean score values in the cemented and cementless groups did not differ significantly (Table **2**).

Radiographic assessment yielded favorable findings in all of the cases. No implant displacement or implant loosening was found. In 3 out of 38 knees in the cementless group and 12 out of 23 knees in the cemented group, RLLs were observed (p < 0.01). RLLs were partial and small (< 1 mm) in all cases and not associated with clinical failure.

One patient in the cemented implant group (3.7%) had revision surgery to replace the patellofemoral joint due to painful arthrosis of this joint six years after the implant surgery. However, this did not affect the original implant. One patient with cementless UKA (2.0%) received revision surgery nearly five months after the initial implant to repair a dislocated meniscal bearing. Similarly, this case did not involve the medial compartment implant. The incidence of complications did not differ significantly between the two groups (p = 1.0).

## 4 DISCUSSION

The most important finding of this study was that both the cemented and cementless Oxford phase 3 implants yielded excellent midterm clinical and functional results. The outcome of the BHT was used to decide whether patients are eligible for cementless UKA. We found that cementless UKA offers shorter operation times and may lead to a lower incidence of postoperative RLLs. The average thickness of the polyethylene insert in cementless group was 0.6 mm thinner than in the cemented group.

A modified titanium porous-coated and hydroxyapatite-coated implant enabled the excellent results of cementless Oxford UKA [11, 14, 15, 22, 23]. Several published papers reflect the increased interest in these implants [11, 15, 24, 25]. We compared the results of cemented and cementless Oxford UKA knee replacement after an approximate seven-year follow-up. Mean scores in the cemented and cementless groups did not differ significantly. Our findings of slightly better follow-up evaluations for the objective KSS, OKS, and WOMAC scores aligned with similar results reported by others [11, 15, 24, 25]. The cementless implant group had an average operating time of 15.5 minutes shorter than the cemented implant group. During the average seven-year follow-up period, we found no loosening of any component of either the cemented or cementless implants. Therefore, taking implant loosening as endpoint of interest, we noted 100% implant survival. One case in the cementless group required patellofemoral replacement, and one case in the cemented implant group required a meniscal insert exchange, but the femoral and tibial components were well fixed. The survival of the implants in both groups seems slightly better than previously published results. However, our small population size (79 cases) may account for this difference. Kerens *et al.* achieved 90% survival after a 34-month follow-up in patients with cementless implants, and 84% survival after a 54-month follow-up in patients with cemented implants [24]. Liddle *et al.* reported a 97.2% survival of cementless Oxford implants at a six-year follow-up [14]. We believe the success of cementless Oxford UKA implants may rely on the intraoperative bone quality assessment and a more sparing tibial plateau resection. The more bone preserving resection of the tibia plateau should create a better condition for cementless UKA.

The major study limitation was that group allocation was based on patient characteristics; hence the outcomes are susceptible to confounding by indication. Another limitation was the relatively small number of cases and shorter follow-up time of the cementless group compared with the cemented group. Also, the number of patients without postoperative radiography was high. Finally, two experienced surgeons in a single institution performed all surgical procedures, so our findings are not readily generalizable.

## CONCLUSION

Both cemented and cementless Oxford phase 3 implants yielded excellent midterm clinical and functional results. The present study was unable to confer significant differences between them. However, cementless UKA offers shorter operation times, and the average thickness of the polyethylene insert in cementless group was 0.6 mm thinner than in the cemented group. In our series of patients, the Bone Hardness Test has been shown to be a simple and useful way of assessing eligibility for cementless UKA. We found that the longevity of both the cementless and the cemented device was similar, and any revisions of the patellofemoral joint or exchange of inserts were not dependent on the type of fixation. As sample size and follow-up time in the current study were limited, further larger studies are required to assess whether cementless UKA reduces the need for revision compared to cemented fixation.

## Figures and Tables

**Table 1:** Baseline characteristics of the study cohorts.

	Cemented (n = 29)	Cementless (n = 50)	p-value
Women (n, %)	27 (93.1)	40 (80.0%)	0.19
Age at surgery [Years]*	65 ± 8.6 (47 to 81)	65.3 ± 7.5 (48 to 79)	0.87
Preoperative OKS*	15 ± 2.9 (12 – 21)	14.7 ± 2.6 (11 – 21)	0.76
KSS knee score*	33.4 ± 11 (14 – 70)	36.3 ± 12 (19 – 70)	0.45
KSS function score*	34 ± 11 (5 – 50)	34.6 ± 9 (5 – 48)	0.66
WOMAC Total*	37.1 ± 5 (20 – 44)	37.8 ± 2.4 (33 – 44)	0.67
WOMAC Pain*	6.1 ± 1.7 (3 - 10)	5.8 ± 1.6 (3 - 11)	0.65
WOMAC Stiffness*	5.2 ± 1.1 (4 – 7)	5.1 ± 1.2 (2 – 7)	0.72
WOMAC Function*	25.8 ± 4.5 (11 – 30)	26.8 ± 1.4 (24 – 30)	0.77

* Presented as mean ± standard deviation (range). Abbreviations: OKS, Oxford Knee Score; KSS, Knee Society Score; WOMAC, Western Ontario and McMaster Universities Osteoarthritis Index.

**Table 2:** Clinical and functional scores at latest follow-up.

	Cemented (n = 29)	Cementless (n = 50)	p-value
OKS*	37.9 ± 3.7 (29 – 46)	38.6 ± 2.7 (32 – 46)	0.67
KSS knee score	86.5 ± 9.5 (58 – 97)	87.5 ± 9.7 (57 – 97)	0.78
KSS function score	73.6 ± 12 (60 – 100)	74.4 ± 13 (65 – 100)	0.75
WOMAC Total	76.6 ± 10 (47 – 93)	78.2 ± 6.3 (56 – 94)	0.63
WOMAC Pain	16.8 ± 2.2 (11 – 20)	17.1 ±1.4 (11 – 20)	0.77
WOMAC Stiffness	6.2 ± 1.0 (5 – 8)	6.2 ± 1-1 (5 – 9)	0.67
WOMAC Function	53.6 ± 7.7 (31 – 66)	54.9 ± 4.4 (40 – 66)	0.87

* Presented as mean ± standard deviation (range). Abbreviations: OKS, Oxford Knee Score; KSS, Knee Society Score; WOMAC, Western Ontario and McMaster Universities Osteoarthritis Index.
